# Evaluation of Perfusion Cell Culture Conditions in a Double-Layered Microphysiological System Using AI-Assisted Morphological Analysis

**DOI:** 10.3390/mi16030327

**Published:** 2025-03-12

**Authors:** Naokata Kutsuzawa, Tomomi Goto, Hiroko Nakamura, Miwa Maeda, Masaki Kinehara, Junko Sakagami, Hiroshi Kimura

**Affiliations:** 1 Micro/Nano Technology Center, Tokai University, 4-1-1 Kitakaname, Hiratsuka 259-1292, Kanagawa, Japan; 2 Division of Pulmonary Medicine, Department of Medicine, Tokai University School of Medicine, 143 Shimokasuya, Isehara 259-1143, Kanagawa, Japan; 3 The Institute of Medical Sciences, Tokai University School of Medicine, 143 Shimokasuya, Isehara 259-1143, Kanagawa, Japan; 4 Nikon Healthcare R&D Center Shonan, Nikon Corporation, Shonan Health Innovation Park, 2-26-1 Muraoka-higashi, Fujisawa 251-8555, Kanagawa, Japan

**Keywords:** microphysiological system, microfluidic chip, Caco-2, Fluid3D-X^®^

## Abstract

In recent years, microphysiological systems (MPS) using microfluidic technology as a new in vitro experimental system have shown promise as an alternative to animal experiments in the development of drugs, especially in the field of drug discovery, and some reports have indicated that MPS experiments have the potential to be a valuable tool to obtain outcomes comparable to those of animal experiments. We have commercialized the Fluid3D-X^®^, a double-layer microfluidic chip made of polyethylene terephthalate (PET), under the Japan Agency for Medical Research and Development (AMED) MPS development research project and have applied it to various organ models. When intestinal epithelial cells, Caco-2, were cultured using Fluid3D-X^®^ and a peristaltic pump, villi-like structures were formed in the microchannels. Still, the degree of formation differed between the upstream and downstream sides. To examine the consideration points regarding the effects of the nutrient and oxygen supply by the chip material and the medium perfusion rate and direction on cells in the widely used double-layer microfluidic chip and to demonstrate the usefulness of a new imaging evaluation method using artificial intelligence technology as an assistive tool for the morphological evaluation of cells, the cell morphology in the channels was quantified and evaluated using the Nikon NIS.ai and microscopic observation. Villi-like structures were predominant upstream of the top channel, independent of the medium perfusion on the bottom channel, and those structures downstream developed with an increased flow rate. Additionally, compared to the Fluid3D-X^®^, the chip made of PDMS showed almost uniform villi-like sterilization in the channel. The result indicates that the environment within the microchannels differs because the amount of nutrients and oxygen supply varies depending on the medium’s perfusion and the material of the chips. As the amount of oxygen and nutrients required by different cell types differs, it is necessary to study the optimization of culture conditions according to the characteristics of the cells handled. It was also demonstrated that the AI-based image analysis method is helpful as a quantification method for the differences in cell morphology in the microchannel observed under a microscope.

## 1. Introduction

In recent years, microphysiological systems (MPSs) utilizing microfluidic technology have been proposed as an alternative to animal experiments, and extensive research has been conducted in the fields of pharmacology and medicine for their practical application [1]. In particular, the MPS, such as organ-on-a-chip (OoC), is an in vitro experimental system in which various cells are cultured using microfluidics to construct physiological organ models and is expected to be an alternative method for animal experiments in drug development, especially in the field of drug discovery [2]. Lately, in Europe, animal testing in cosmetics development has already been prohibited [3], and the US Food and Drug Administration promulgation of the Modernization Act 2.0, which will not require animal testing before clinical trials by the end of 2022, has also accelerated the consideration of the practical application of MPSs for research use [4,5]. MPSs have been proposed and widely used for various organs and tissues, including the liver, intestine, kidney, and blood–brain barrier [6].

Based on this background, various MPS devices and chips have been developed nowadays, some of which have been launched commercially and are being placed into practical use. There are several reports that can replace some fields of animal experiments, especially ADME/toxicity tests, even though they are not in standard format [7,8,9,10]. In particular, similar products have been marketed since the double-layer channel-type device marketed by Emulate can be adaptable to various membranous organs [11]. Although each product has an application note for practical use, they are not all related to the cells that the user wants to use. Therefore, it is necessary to consider the culture conditions for each cell to be cultured in the chip.

One of the most typical fields in which the MPS is used is gut-on-a-chip (GoC), which simulates the intestinal tract. GoC can imitate the environment in the intestinal tract, such as the interaction between various cells and the intestinal microbiota, especially dynamic aspects of the intestine, like peristalsis and the transfer of food from the mouth to the anus, which could not be achieved with cell culture inserts previously [6]. This process has made it possible to evaluate the absorption and permeability of food components and drugs, as well as the effects of therapeutic targets and drugs in an in vitro model of inflammatory bowel disease using inflammatory cytokines under conditions more similar to biological conditions. A cell culture with GoC has been reported to extend the cell culture period [12,13]. In addition, it has been reported that applying shear stress, such as by perfusion of the medium, promotes the formation of villus-like structures [14]. In fact, when intestinal epithelial cell line Caco-2 cells were cultured under a medium perfusion system using Fluid3D-X^®^, a double-layer channel-type device that we commercialized in the Japan Agency for Medical Research and Development (AMED) MPS development research project [15], villi-like structures that do not develop in static culture were formed. In contrast, differences in the degree of formation of villi-like structures were observed between the upstream and downstream sides of the microchannel. However, we have not been able to establish a quantification method for the differences in cell morphology within the flow channels that can be recognized microscopically, nor have we been able to examine whether these differences affect the subsequent assay system.

In this study, we investigated the optimal conditions for culturing intestinal epithelial cells in Fluid3D-X^®^, aiming to examine the consideration points concerning the effects on cells of nutrient and oxygen supply by the material and medium flow in the widely used double-layer channel-type chips and to demonstrate the usability of a new imaging evaluation method using artificial intelligence (AI) technology for a morphological evaluation of cells in the channel.

## 2. Materials and Methods

### 2.1. Microfluidic Chip and Perfusion Setup

Two types of microfluidic chips made of different materials were used in this study.

**Figure 1:**
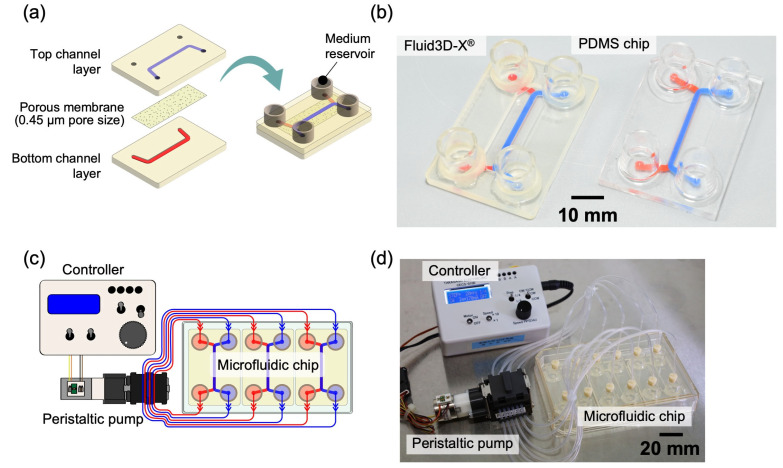
Experimental setup. Microfluidic chip and perfusion system by Takasago Denki. (**a**) Configuration of microfluidic chips, including Fluid3D-X^®^ and PDMS chip. (**b**) Photographs of microfluidic chips: Fluid3D-X^®^ made of PET (**left**) and PDMS chip made of PDMS (**right**); scale bars are 10 mm. (**c**) Schematic diagram of the perfusion culture system using microfluidic chips. (**d**) Actual setup of the perfusion culture system with Fluid3D-X^®^. PDMS: polydimethylpolysiloxane.

The first is Fluid3D-X^®^, a product of Tokyo Ohka Kogyo (Kanagawa, Japan), made of a polyethylene terephthalate (PET). It has a typical double-layer microchannel structure, separated by a porous membrane with a pore size of 0.45 µm and medium reservoirs in each port (Figure 1a) [15]. The microfluidic channel in the cell culture section of Fluid3D-X^®^ has a width of 2 mm, a height of 0.7 mm, a length of 32 mm, and an effective culture area of 64 mm^2^. The chip has five dots beside the microchannel to serve as imaging landmarks.

The second is a microfluidic chip made of polydimethylpolysiloxane (PDMS) with the same geometry as the Fluid3D-X^®^ that we made. The PDMS chip was fabricated using the previously reported fabrication process [16]. Briefly, PDMS layers with microchannel structures were fabricated by soft lithography. Polymethyl methacrylate master molds for soft lithography were made by a 3D modeling machine (MODELA MDX-50, Roland, Shizuoka, Japan). A 10:1 mixture of unpolymerized PDMS (SILPOT184; Dow Toray, Tokyo, Japan) and catalyst was poured into the master mold and baked in an oven at 75 °C for 2 h. For permanent bonding to the PDMS layers, the surface of the porous membrane was coated with 6.7% (*v*/*v*) 3-aminopropyltriethoxysilane solution (KBE-903, Shin-Etsu Chemical, Tokyo, Japan) and 2.5% (*v*/*v*) glutaraldehyde solution (17026-32, Kanto Chemical, Tokyo, Japan). Holes (4 mm in diameter) were made in the PDMS layer on the upper microchannel side using a biopsy punch (BP-40F, Kai Medical, Gifu, Japan) at the inlet and outlet ports of the top- and bottom-layer microchannels. The PDMS chip was assembled by sandwiching the porous membrane between the plasma-treated PDMS layers.

The PDMS chip was used in our experiments in the hope that it would provide a higher oxygen supply to the cells than the PET-made Fluid3D-X^®^. Hereafter, we term “Fluid3D-X^®^” the microfluidic chip made of PET (Figure 1b left), and “PDMS chip” the chip with the same configuration as the Fluid3D-X^®^ made of PDMS (Figure 1b right).

To perfuse the culture medium, medium chambers on three microfluidic chips in an ANSI/SLAS-compliant rectangular plate were connected to a peristaltic pump (AQ-RP6R-001, Takasago Fluidic Systems, Nagoya, Japan) with silicone tubes (Figure 1c,d). A controller of the peristaltic pump arbitrarily controlled the flow rate of the medium perfusion. This system could perfuse the medium at the same rate as all the chambers. On the other hand, another pump system (Microtube pump system, Icomes Lab, Iwate, Japan, https://www.icomes.co.jp/en/) could perfuse the medium at the different speeds and directions of each microchannel. We used these two systems for the different cell culture system setups.

### 2.2. Cell Culture Method Using Microfluidic Chips

Caco-2 cells were obtained from the American Type Culture Collection (HTB-37, ATCC, Manassas, VA, USA) and cultured in Dulbecco’s Modified Eagle Medium (DMEM) (12320-032, Thermo Fisher Scientific, Waltham, MA, USA) containing 10% Fetal Bovine Serum (FBS) (10270106, Gibco, Thermo Fisher Scientific), MEM Non-Essential Amino Acids solution (11140-050, Thermo Fisher Scientific) and a Penicillin–Streptomycin–Amphotericin B Suspension (161-23181, FUJIFILM Wako Pure Chemical Corporation, Osaka, Japan).

**Figure 2:**
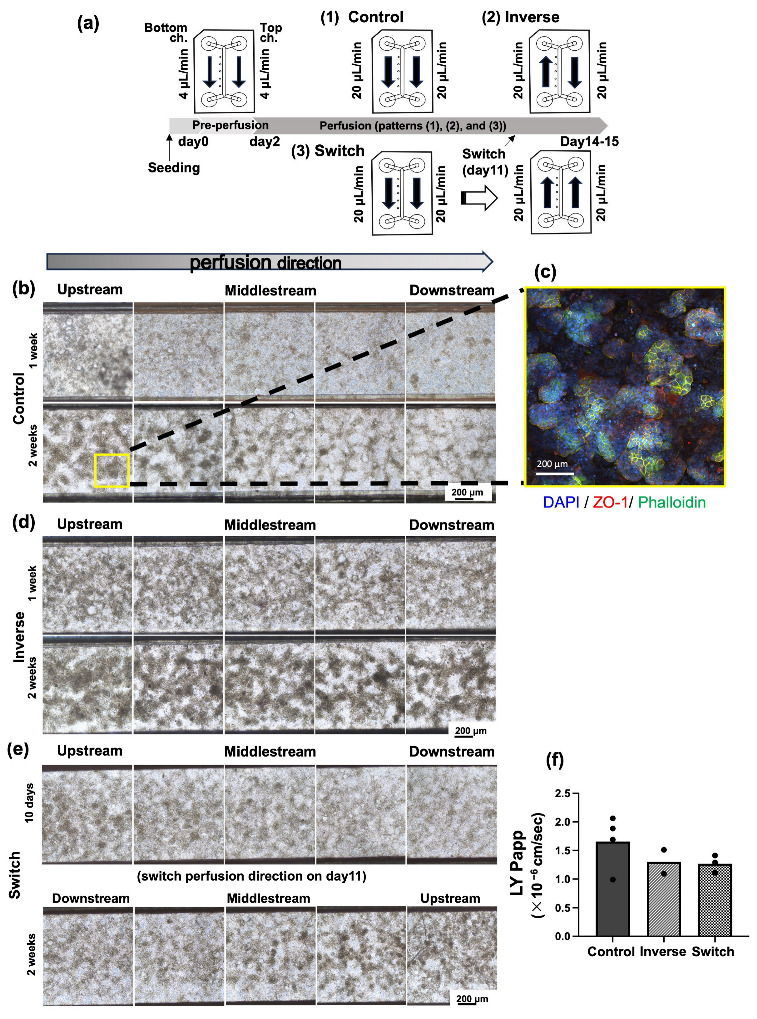
Representative data of the cell morphology in the Fluid3D-X^®^ microchannel under several conditions of perfusion directions. (**a**) Schematic diagram of the experimental schedule. (1) Control: after pre-perfusion (4 μL/min for 2 days), the culture medium of the top and bottom channels was perfused toward the same direction at the 20 μL/min rate. (2) Inverse: after pre-perfusion, the culture medium of the top and the bottom channels was perfused in opposite directions at the rate of 20 μL/min. (3) Switch: after pre-perfusion, the culture medium of the top and the bottom channels was perfused toward the same direction at the rate of 20 μL/min until day 11, then we switched the perfusion direction at the rate of 20 μL/min. A cell culture was conducted for 2 weeks (14 ± 1 days) after seeding. (**b**) Images of a representative bright field of the Control at 1 week (7 ± 1 days) and 2 weeks (14 ± 1 days). Magnification ×40, scale bar 200 μm. (**c**) Image of immunocytochemistry of villi-like structure. Magnification ×200, scale bar 200 μm. (**d**) Images of a representative bright field of ‘Inverse’ at 1 week (7 ± 1 days) and 2 weeks (14 ± 1 days). Magnification ×40, scale bar 200 μm. (**e**) Images of a representative bright field of ‘Switch’ at 10 days and 2 weeks (14 ± 1 days). Magnification ×40, scale bar 200 μm. (**f**) The result of the Lucifer Yellow permeability test. Values were the means of the *P_app_* of each chip, and each dot indicates one chip. ZO-1: Zonula occludens-1; DAPI: 4′,6-diamidino-2-phenylindole; LY: Lucifer Yellow.

We cooled the microfluidic chips to 4 °C before the extracellular matrix coating. Then, 110 μL of the Matrigel^®^ Matrix Basement Membrane (356237, Corning, Corning, NY, USA) diluted 30× in FBS-free DMEM was applied to the top and bottom channels of the chip, and microfluidic chips were incubated at 4 °C until the next day. The Caco-2 cells cultured on the Petri dish covered around 90% and were washed twice with PBS and then treated with Trypsin (T4049, Sigma-Aldrich, St. Louis, MO, USA). The cells were suspended to 2 × 10^6^ cells/mL (2 × 10^5^ cells/cm^2^), and 110 μL of the cell suspension was seeded onto the top channel with a micropipette fitted with a wide-pore tip. Subsequently, 120 µL of DMEM was injected into the bottom channel, and the microfluidic chips in the plate were placed in a CO_2_ incubator (37 °C, 5% CO_2_) to allow the cells to attach to the membrane. After 4 to 5 h, when cell attachment was confirmed, the peristaltic pump was equipped to the perfusion system, and 650 μL of DMEM was added to the medium reservoirs of both the inlet and outlet simultaneously with a multipipette from the top to bottom channels. After filling the medium on the reservoirs, medium perfusion, whose speed was about 4 µL/min, was performed (pre-perfusion). On day 2, the medium was changed, the perfusion speed was increased, and directions were modified for each condition. The medium was changed every 2 or 3 days (Figure 2a).

### 2.3. Lucifer Yellow Permeability Test

Before the permeability test, the medium in the channels and inlet and outlet reservoirs were replaced with transport buffer (TP buffer, HBSS with Ca^2+^, Mg^2+^, containing 10 mM HEPES, pH 7.4, phenol-red-free, FUJIFILM Wako Pure Chemical Corporation). First, we removed the medium in both the inlet and outlet reservoirs with a multipipette and added 600 µL of TP buffer onto the inlet reservoir. This process was repeated twice, and then the chips were incubated under 37 °C 5% CO_2_ for 30–60 min. After acclimation, TP buffer in both the inlet and outlet reservoirs was removed with a multipipette, and 500 µL of 100 µM Lucifer Yellow (LY, 128-06271, FUJIFILM Wako Pure Chemical Corporation) in TP buffer was applied onto the inlet reservoir on the top channel. To ensure a uniform distribution of LY in the channel, 200 µL of the LY solution was collected from the reservoir on the outlet side and returned twice to the inlet. The same procedure was performed with 500 µL of TP buffer for the bottom channel. Then, 100 µL of the solution was collected from the top and bottom outlets and served as samples at 0 h. Chips were incubated under 37 °C 5% CO_2_. Furthermore, 30 min later, the same procedure as 0 h was performed, and 100 µL of the solution collected from the top and bottom outlets were served as samples at 0.5 h.

The samples collected at each time point were measured with a microplate reader (SH-9500Lab, CORONA ELECTRIC, Ibaraki, Japan) at Ex/Em: 485 nm/538 nm. The transmission coefficient was calculated using the following formula:

(1) $$P_{app}=\frac{dQ}{dt}\cdot \frac{1}{C_{0}\cdot S}$$

where *P_app_* (cm/s) is the transmission coefficient, *dQ*/*dt* (nmol/s) is the transmission velocity, *C*_0_ is the initial concentration of the LY on the apical side, and *S* is the surface area of the porous membrane.

### 2.4. Imaging and Analysis

#### 2.4.1. Immunocytochemistry

To detect the Zonula Occuludens-1 (ZO-1) and phalloidin protein expression in Caco-2 cells, they were cultured in Fluid3D-X^®^ and fixed using 4% paraformaldehyde (PFA, FUJIFILM Wako Pure Chemical Corporation). After blocking with 1% bovine serum albumin in PBS, the cells were incubated overnight with rabbit anti-ZO-1 antibody (Proteintech Group, Inc., Rosemont, IL, USA). Next, the cells were incubated with the secondary antibody Alexa Fluor 568 donkey anti-rabbit IgG (Invitrogen, Carlsbad, CA, USA) for 1 h. Subsequently, Acti-Stain 488 Fluorescent Phalloidin (Cytoskelton. Inc., Denver, CO, USA) and DAPI solution (DOJINDO LABORATORIES, Kumamoto, Japan) were added. Finally, the cells were mounted in the Vectashield medium (Vector Laboratories, Burlingame, CA, USA).

Cell imaging was performed using a confocal microscope (A1R, Nikon Corporation, Tokyo, Japan) at the Tokai University Imaging Center for Advanced Research.

#### 2.4.2. AI-Assisted Morphological Analysis

Z-stack bright-field imaging was performed using an inverted microscope (ECLIPSE Ti-E, Nikon) equipped with a 10× objective lens (PLAN APO λD, Nikon Corporation), a halogen lamp house (D-LH/LC, Nikon Corporation) for bright-field transillumination, and a camera (ORCA-Flash4.0, Hamamatsu Photonics, Shizuoka, Japan). The Z-stack imaging covered a range of 300 μm with a step size of 5 μm (Figure S1a). Subsequently, these Z-stack images were processed using the volume contrast (VC) function of the NIS-Elements software (Nikon Corporation) to generate VC images. VC imaging, based on quantitative phase imaging (QPI) principles—a non-invasive and label-free method that measures the phase shift of light passing through transparent samples—has emerged as a powerful method for investigating the morphology and thickness of cells and tissues [17,18]. This approach is particularly advantageous for real-time observations of the dynamic cellular processes without altering their physiology. Additionally, the built-in Extended Depth of Focus (EDF) function of the NIS-Elements software ver. 6.01.00 was used to project these Z-stack images into a single focused image by selecting the focused regions. In the next step, the concert.ai learning feature of the NIS.ai module in NIS-Elements—an AI-powered function designed to enhance image analysis— was utilized to train the AI model. The AI model was trained using six sets of cropped EDF and VC images from the flow channel regions of the microfluidic chips (Figure S1b). Finally, the AI-VC images were generated by the trained convert.ai model from the EDF image of the stitched whole image of the microfluidic chips (Figure S1c). The generated AI-VC images were then cropped into three regions and analyzed using the General Analysis 3 (GA3) function of the NIS-Elements software.

## 3. Results

### 3.1. Alteration of Cell Morphology Depending on the Direction of Medium Perfusion

In the control group, in which the flow rate was approximately 20 μL/min after gentle pre-perfusion (4 μL/min) until day 2 (Figure 2a; Control), it was observed that the chorionic villi-like structures (Figure 2b,c, and Figure S2c) were highly advanced in the upstream (the inlet side) of the flow channel. Those structures became sparser toward downstream (the outlet side) (Figure 2b). In the investigation in which the direction of medium perfusion in the top and bottom channels was reversed (top channel: from the inlet to the outlet; bottom channel: the outlet to the inlet, Figure 2a; Inverse), regardless of the direction of medium perfusion in the bottom channel, villi-like structures were observed in the upstream side of the top channel in the bright-field image. Villi-like structures slightly decreased toward the downstream side; however, more villi-like structures were observed than in the control group through the top channel (Figure 2d).

Next, after perfusing the medium in the same direction (both top and bottom channels: perfused from the inlet to the outlet) until day 11, the medium was perfused in the opposite direction in both the top and bottom channels (both top and bottom channels: perfused from the outlet to the inlet, Figure 2a; Switch). The inlet side, which had been the upstream side until day 11, lost its villi-like structures over time, and the formation of villi-like structures progressed on the inlet side, which had newly become the upstream side (Figure 2e).

In the LY permeability test of these three investigations (Control, Inverse, Switch), the *P_app_* of each condition was less than 2.0 × 10^−6^ cm/s, and there was no difference among the three conditions (Figure 2f).

### 3.2. Alteration of Cell Morphology Depending on the Flow Rate of Medium Perfusion

**Figure 3:**
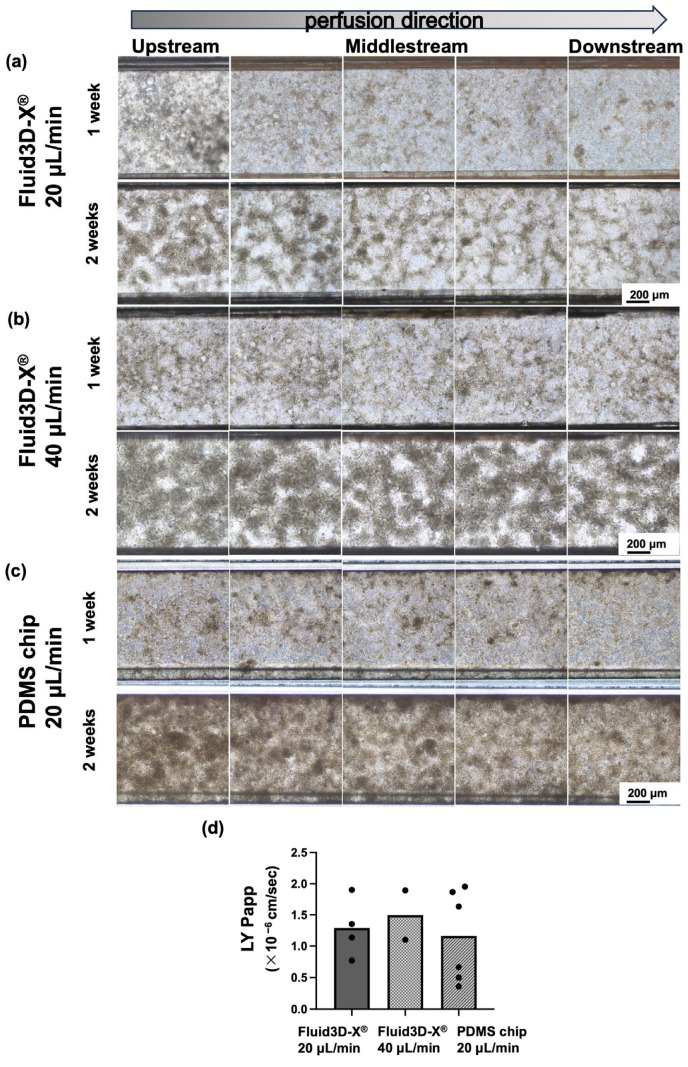
Representative data of the cell morphology in the microchannel under several culture conditions. (**a**) Images of the representative bright field of control (chip: Fluid3D-X^®^, perfusion rate: 20 μL/min after pre-perfusion) at 1 week (7 ± 1 days) and 2 weeks (14 ± 1 days). (**b**) Images of the representative bright field of doubled speed (chip: Fluid3D-X^®^, perfusion rate: 40 μL/min after pre-perfusion) at 1 week (7 ± 1 days) and 2 weeks (14 ± 1 days). (**c**) Images of the representative bright field of control (chip: PDMS chip, perfusion rate: 20 μL/min after pre-perfusion) at 1 week (7 ± 1 days) and 2 weeks (14 ± 1 days). Left: upstream; middle: middle; right: downstream of chip. Magnification ×100, scale bar 400 μm. (**d**) The result of the Lucifer Yellow permeability test. Values were the means of the *P_app_* of each chip, and each dot indicates one chip. LY: Lucifer Yellow; PDMS: polydimethylpolysiloxane.

In the control (Fluid3D-X^®^, flow rate; 20 μL/min), as mentioned above, the formation of villi-like structures was more advanced upstream of the top channel and less advanced downstream in the bright-field images (Figure 3a). In contrast, in the group in which the flow rate was increased to 40 μL/min, more villi-like structures were observed upstream of the top channel in the bright-field image as in the control group. However, compared to the control group, more villi-like structures were observed in the midstream and downstream of the channel, although each was thinner than that of the upstream side (Figure 3b). Additionally, the flow rate was further increased to 60 µL/min, which was observed to be not much different from the flow rate of 40 µL/min under a conventional optical microscope (Figure S2a). Immunocytochemistry was also performed, and the villi-like structures were observed equally in the upstream, midstream, and downstream areas (Figure S2b).

**Figure 4:**
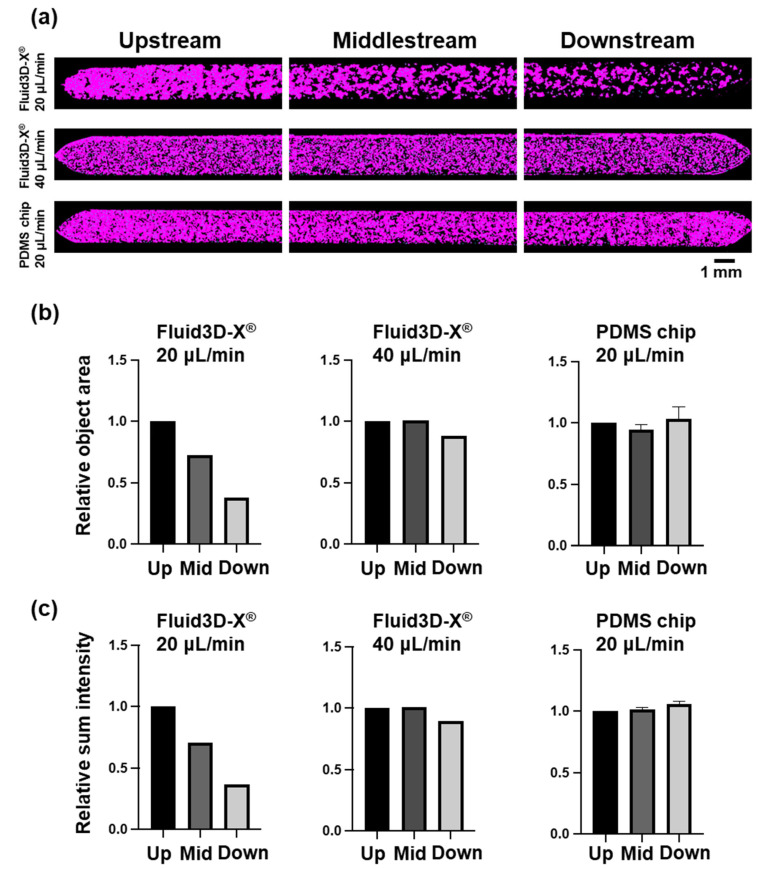
The 3D structure distribution in a microfluidic channel quantified by NIS.ai. (**a**) Images of the microchannels with three different conditions by AI-VC. The whole microchannel was divided into three regions: upstream (**left**), middlestream (**center**), and downstream (**right**). Top: Fluid3D-X^®^, perfusion rate of 20 μL/min; middle: Fluid3D-X^®^, perfusion rate of 40 μL/min; bottom: PDMS chip, perfusion speed of 20 μL/min. Scale bar 1 mm. (**b**) Relative total object area of AI-VC. (**c**) Relative Sum Intensity of AI-VC. Each parameter was standardized by its ‘upstream’ value. Left: Fluid3D-X^®^, perfusion rate of 20 μL/min; center: Fluid3D-X^®^, perfusion rate of 40 μL/min; right: PDMS chip, perfusion rate of 20 μL/min. Up: upstream; Mid: middlestream; Down: downstream.

We attempted to quantify the morphological changes seen in these bright-field images and immunocytochemistry using NIS.ai. We evaluated the area ratio of the villi-like structures to the flow channel area and the thickness of the cells by VC intensity, i.e., the development of villi-like structures, with NIS.ai. The results showed a decrease in the total object area and a Sum Intensity from upstream to downstream in the control group, and the quantitative analysis showed similar changes to those observed in the bright-field images (Figure 4). In contrast, the total object area and Sum Intensity of the AI-VC were almost the same in the group in which the flow rate was increased to 40 μL/min, with a very slight decrease downstream (Figure 4).

The results of the LY permeability test showed no difference, with a *P_app_* less than 2.0 × 10^−6^ cm/s under different flow rates (Figure 3d).

### 3.3. Alteration of Cell Morphology Depending on the Material of Chips

The Caco-2 cell cultures in the Fluid 3D-X^®^ showed higher levels of villi-like structure formation upstream of the top channel in the bright-field images and lower levels downstream, as described above, and the results quantified by NIS.ai were similar. In contrast, the Caco-2 cells cultured on the PDMS chip showed a uniform villi-like structure from upstream to downstream in the bright-field images (Figure 3c). We evaluated the thicknesses of the cells in the channels and the development of villi-like structures by the area ratio of spatially developed structures to the channel area and VC intensity using NIS.ai by using the same method as for the medium perfusion rate study. The results showed that the total object area and Sum Intensity of the AI-VC did not decrease from upstream to downstream (Figure 4b right, and Figure 4c right), indicating that the villi-like structures were formed almost uniformly from upstream to downstream.

The results of the LY permeability test showed that the *P_app_* was less than 2.0 × 10^−6^ cm/s under different material conditions, showing no difference compared to the control group (Figure 3d).

## 4. Discussion

In this study, we used the Fluid3D-X^®^ [15], a double-layer MPS chip, to investigate the effects of the chip’s materials and the medium’s flow rate and direction on the supply of nutrients and oxygen to cells.

Caco-2 cells are widely used in an in vitro model of the intestinal tract to study nutrient and pharmaceutical permeability, physiological processes, and pathological mechanisms [19]. It has been reported that it takes about three weeks for Caco-2 cells to function as intestinal epithelium when cultured on cell culture inserts [20]. In contrast, it has also been reported that Caco-2 cells form villi-like structures and provide a barrier function against small molecules when cultured in a microfluidic device with medium perfusion, which is a favorable condition compared to static cell culture inserts [21,22]. The epithelial barrier robustness was guaranteed regardless of the cell morphology in the LY permeability test under the various conditions in this study.

We considered three factors regarding the morphological characteristics of the Caco-2 cells focused on in this study. The first point is the effect of the morphogen secreted by the cells. In a study using a double-layer chip by Valiei et al. in which Caco-2 cells were cultured with medium perfusion, there was a high degree of villi-like structures upstream of the channel but less downstream. They reported that Caco-2 cells secrete Dickkopf-1 (DKK-1), an antagonist of Wnt, to the basolateral side, and that the perfusion of the medium in the bottom channel inhibited the formation of villi-like structures on the downstream side where the DKK-1 concentration was increased [23]. It has also been reported by Shin et al. that the perfusion of the basal medium is important for the morphogenesis of intestinal epithelial cells through the use of a microfluidic device that can perfuse the medium on the basal side of the cell culture insert [24].

The second point is the change in nutrient and oxygen supply depending on the chip material and the amount of media perfusion. In this study, microfluidic chips made of PDMS that had high oxygen permeability formed villi-like structures almost uniformly from upstream to downstream. We cannot find the study that reports the formation of villi-like structures in intestinal epithelial cell cultures on microfluidic chips, which vary the distribution of villi-like structures in microfluidic channels. This may be because the microfluidic chip material is PDMS [25,26], which has higher oxygen permeability and thus did not cause a hypoxic environment, especially in the flow channel, or may have caused non-uniformity in oxygen concentration. Fluid3D-X^®^, on the other hand, was made of PET, which has less drug adsorption and sorption [15]. The gas permeability of PET is extremely low compared to PDMS. A comparison of the Fluid3D-X^®^ and PDMS chip used in this study also showed apparent differences in cell morphology in the channels with the same amount of media perfusion, suggesting that the difference in oxygen supply had an effect. In addition, the more developed villi-like structures downstream with increased medium perfusion in the flow rate study using Fluid3D-X^®^ may be attributed to the fact that oxygen and nutrients were supplied to the cells further downstream in the flow channel.

The third point is the imposition of the shear stress by perfusion of the medium. Pocock et al. cultured Caco-2 cells using a gut-on-a-chip made of PDMS with or without medium perfusion and reported that villi-like structures developed when the medium was perfused [27]. In the report, the expression of F-actin was upregulated in the villi-like structures, and F-actin was formed from the apical to the basal side. Since this study was performed on PDMS chips, which have high oxygen permeability, it is suggested that the changes may be due to the shear stress caused by perfusion of the medium. The perfusion rate of the medium was also examined in the study by Valiei et al., which showed that at flow rates in the appropriate range, higher flow rates resulted in a rapid and higher formation of villi-like structures, but at flow rates outside of that appropriate range, the formation of villi-like structures was delayed [23]. On the other hand, we cannot rule out the possibility that these differences in outcomes due to the medium perfusion are attributed to the supply of nutrients and oxygen by the medium perfusion. For instance, in the aforementioned morphological changes induced by the Wnt signaling pathway, it has been reported that DKK-1 expression is enhanced in glioma cells [28] and bone [29] when the partial pressure of oxygen is decreased, and it is possible that differences in oxygen conditions caused by the difference of the materials of chips or perfusion rate of the medium, therefore, affected DKK-1 secretion.

While there are cells such as Caco-2 that show morphological differences in response to differences in the cell culture environment, it is reported that iPS cell-derived intestinal epithelial cells, F-hiSIEC^TM^, cultured in Fluid3D-X^®^, showed uniform cell morphology throughout the microchannel without differences [15]. It is a fact that some types of cells show no morphological changes while others have a non-uniform cell morphology in the microchannel due to the changes in oxygen permeability and nutrient distribution caused by the perfusion rate of the medium and the materials of the chip. Consequently, optimization of the culture environment may or may not be necessary depending on the type and material of the MPS chip and the cells. Therefore, it will be necessary to consider these factors depending on the cell type and culture method. Conversely, it is also possible to actively use a culture environment that changes gradually from upstream to downstream. For instance, Matsumoto et al. successfully reproduced liver zonation in liver-on-a-chip by taking advantage of the oxygen concentration distribution [30]. Elsewhere, Shah et al. achieved a co-culture of Caco-2 cells and enterobacteria utilizing a gradient of oxygen concentration in the HuMix device—a double-layer channel device [31].

In recent years, the application of AI technology for cell morphological evaluations has been promoted in research using MPS. In airway-on-a-chip studies using human bronchial epithelial cells, the culture period until the cells can be used for assays is approximately 30 days, which is a long time, and there is a possibility of culture failure during the culture process, as well as time and cost losses if the cells are not in a suitable condition for the assay. Accordingly, it has been reported that a culture success prediction system using AI technology is useful to ensure subsequent culture and cell conditions in the early stages of a culture [32]. Han et al. reported technology for culturing human iPSC-derived sensory neurons in MPS chips and its application to screening for drug-induced neuropathy caused by anticancer drugs using AI-based deep learning [33]. In this study, we used NIS.ai to quantify the villi-like structures of Caco-2 cells in the microchannel using several indices, including the area ratio of villus-like structures to the channel area and the intensity of VC. Using this methodology, we have shown that it is possible to quantify the sparsening of the villi-like structures from upstream to downstream in the channel, as observed microscopically. Also, it was found that cell morphology can be more accurately quantified from image data by using AI learning to complement missing data in regions where image analysis is difficult due to the scattering of transmitted light in bright-field images. Consequently, image analysis using AI technology is very effective as a supporting technology in research using MPS, and it is expected to be a new technique for evaluating cell morphology in cell cultures using MPS in the future.

The limitation of this study is focused on the morphological characteristics, and the molecular mechanism has not been examined, which is an issue for further study.

## 5. Conclusions

In this study, we discussed the morphological heterogeneity observed in Caco-2 cells cultured using Fluid3D-X^®^, a double-layer-type MPS chip, focusing on the perfusion conditions of the medium and the oxygen permeability of the chip material. This study shows that the cell culture environment is not uniform in the channel under certain conditions and reveals the importance of its optimization. On the other hand, when human iPS cell-derived intestinal cells (F-hiSIEC^TM^, FUJIFILM) were cultured using Fluid3D-X^®^, no morphological heterogeneity was observed under the culture conditions conducted in this study [15]. Since the oxygen and nutrient requirements differ depending on the cell type, it is necessary to consider optimizing the culture conditions according to the characteristics of the cells cultured in the MPS chips. It is expected that by optimizing culture conditions and constructing organ models, MPS will be able to accurately predict pharmacokinetics and safety in humans, which have been difficult to predict with conventional in vitro experimental systems, and thus will serve as an alternative method for animal experiments.

## Data Availability

The original contributions presented in this study are included in the article/Supplementary Materials. Further inquiries can be directed to the corresponding author.

## Supplementary Materials

The following supporting information can be downloaded at: https://www.mdpi.com/article/10.3390/mi16030327/s1, Figure S1: Cell morphology imaging in the microfluidic channel and image analysis method using NIS.ai; Figure S2: Cell morphology image in the microfluidic channel.
