# Supplementary material for: Evaluation of Perfusion Cell Culture Conditions in a Double-Layered Microphysiological System Using AI-Assisted Morphological Analysis
Source: Micromachines (Basel). 2025 Mar 12;16(3):327. doi: 10.3390/mi16030327 (PMC11945015; doi:10.3390/mi16030327)
Supplement: Supplementary file 1 [file micromachines-16-00327-s001.zip › micromachines-3503353-supplementary.pdf]

Supplementary

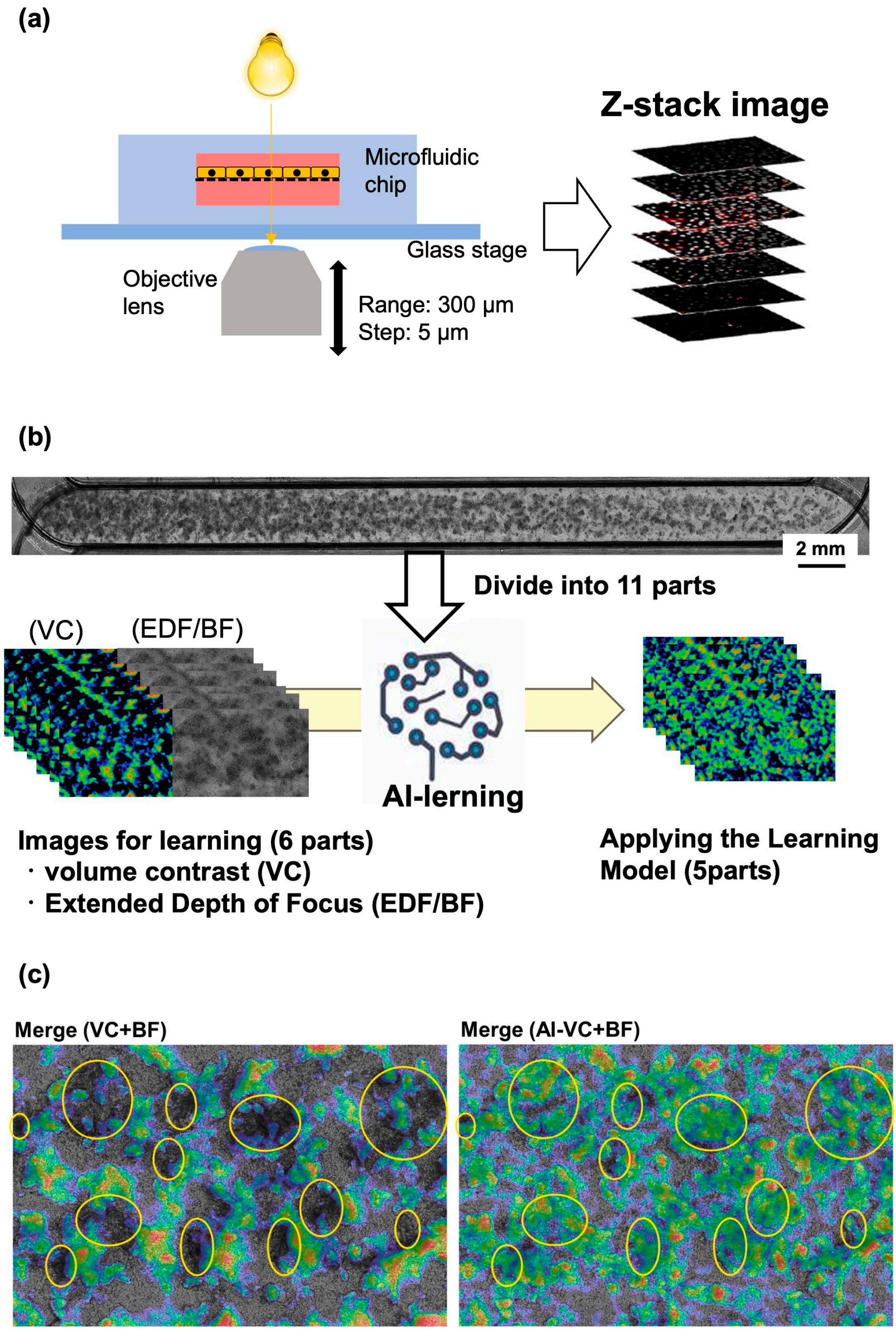

Figure S1. Cell morphology imaging in the microfluidic channel and image analysis method using NIS.ai. (a) Schematic diagram of VC imaging technique for an entire microfluidic channel. (b) Upper: BF image of the whole microfluidic channel. Lower: Schematic diagram of AI learning models and application of AI learning models. Scale bar; 2 mm. (c) Image of the non-AI-assisted image of VC+BF (merged, left) and AI-assisted image (merged, right). The black areas of the VC+BF image (indicated by a yellow circle) are areas where transmitted light was scattered, indicating that VC conversion was unavailable from the BF image. AI-assisted image (AI-VC+BF) allows for more

accurate evaluation with complementary information on the same site. The procedure is mentioned in *2.4.2 AI-Assisted Morphological Analysis*. AI; artificial intelligence, BF; bright field, EDF; Extended Depth of Focus, VC; volume contrast.

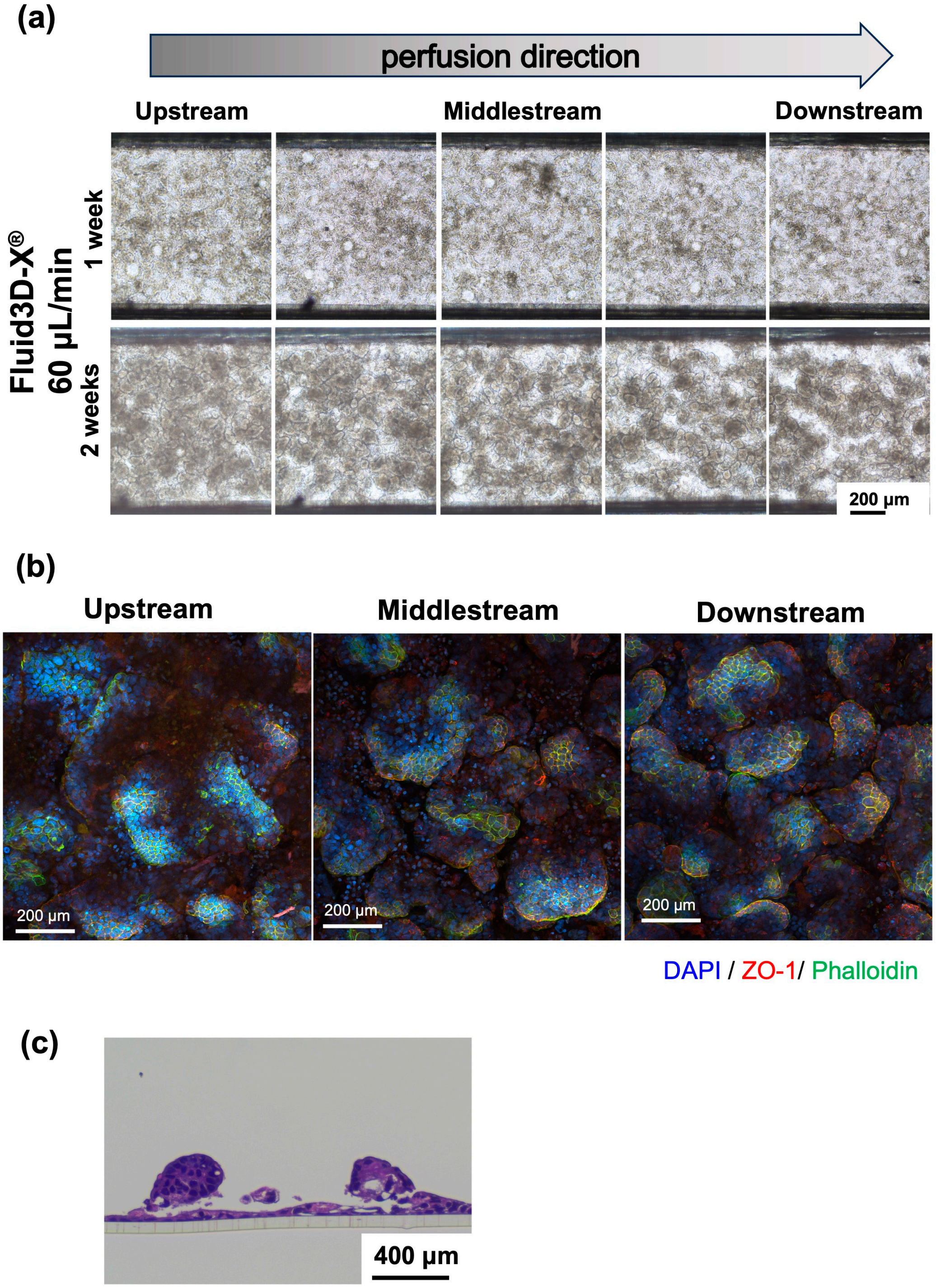

Figure S2. Cell morphology image in the microfluidic channel. (a) Images of the representative bright field of tripled flow rate (chip: Fluid3D-X®, perfusion rate: 60 µL/min after pre-perfusion) at 1 week (7±1 days) and 2 weeks (14±1 days). Magnification ×40, scale bar 200µm. (b) Image of the representative immunocytochemistry of tripled flow rate. Magnification ×200, scale bar 200µm. Blue; DAPI, red; ZO-1, green; Phalloidin. (c) The representative HE

staining. Chip; Fluid3D-X®, flow rate: 20 μL/min after pre-perfusion. Caco-2 cells have villi-like structures on the porous membrane. Magnification ×200, scale bar 400 μm. Preceding HE staining, Caco-2 cells cultured in Fluid3D-X® were fixed by 4% paraformaldehyde (PFA). The membrane of the flow channel was then cut from the Fluid3D-X® using a scalpel and paraffin-embedded. Then, the embedded sample was sectioned by microtome, and slides were deparaffinized and rehydrated before HE staining. ZO-1; Zonula occludens-1, DAPI; 4',6-diamidino-2-phenylindole, HE: Hematoxylin and Eosin.
